# Noninvasive measurement of cardiac stroke volume using pulse wave velocity and aortic dimensions: a simulation study

**DOI:** 10.1186/1475-925X-13-137

**Published:** 2014-09-19

**Authors:** Charles F Babbs

**Affiliations:** Weldon School of Biomedical Engineering, Purdue University, 206 South Martin Jische Drive, West Lafayette, Indiana 47907-2032 USA

## Abstract

**Background:**

Concerns about the cost-effectiveness of invasive hemodynamic monitoring in critically ill patients using pulmonary artery catheters motivate a renewed search for effective noninvasive methods to measure stroke volume. This paper explores a new approach based on noninvasively measured pulse wave velocity, pulse contour, and ultrasonically determined aortic cross sectional area.

**Methods:**

The Bramwell-Hill equation relating pulse wave velocity to aortic compliance is applied. At the time point on the noninvasively measured pulse contour, denoted t_h_, when pulse amplitude has fallen midway between systolic and diastolic values, the portion of stroke volume remaining in the aorta, and in turn the entire stroke volume, can be estimated from the compliance and the pulse waveform. This approach is tested and refined using a numerical model of the systemic circulation including the effects of blood inertia, nonlinear compliance, aortic tapering, varying heart rate, and varying myocardial contractility, in which noninvasively estimated stroke volumes were compared with known stroke volumes in the model.

**Results:**

The Bramwell-Hill approach correctly allows accurate calculation of known, constant aortic compliance in the numerical model. When nonlinear compliance is present the proposed noninvasive technique overestimates true aortic compliance when pulse pressure is large. However, a reasonable correction for nonlinearity can be derived and applied to restore accuracy for normal and for fast heart rates (correlation coefficient > 0.98).

**Conclusions:**

Accurate estimates of cardiac stroke volume based on pulse wave velocity are theoretically possible and feasible. The precision of the method may be less than desired, owing to the dependence of the final result on the square of measured pulse wave velocity and the first power of ultrasonically measured aortic cross sectional area. However, classical formulas for propagation of random errors suggest that the method may still have sufficient precision for clinical applications. It remains as a challenge for experimentalists to explore further the potential of noninvasive measurement of stroke volume using pulse wave velocity. The technique is non-proprietary and open access in full detail, allowing future users to modify and refine the method as guided by practical experience.

## Background

Monitoring cardiac pump function is extremely useful in critical care medicine and has been the standard of care in order to ensure tissue oxygenation [1]. Clinical measurement of cardiac output is traditionally done using a flow-directed Swan-Ganz catheter, placed percutaneously into the pulmonary artery. Typically the pulmonary artery catheter is equipped with an injection port and a downstream temperature sensor to measure total pulmonary artery blood flow by thermodiluiton [2]. In recent years, however, the cost effectiveness of this invasive procedure has been called into question [3–5], especially given complications that occur in about 10 percent of cases [4]. In the SUPPORT trial the 30 day survival critically ill patients was greater in 3552 patients managed without a pulmonary artery catheter than in 2184 similar patients managed with a pulmonary artery catheter [3]. Moreover, in 1008 matched pairs of patients managed either with or without pulmonary artery catheters, the total cost was 38% ($13,600) greater when pulmonary artery catheters were used to monitor heart function. Hence it is timely to revisit the issue of clinical monitoring of stroke volume and cardiac output with an eye toward less invasive and less expensive options.

According to Geerts, Aarts, and Jansen [6] the ideal technique for the measurement of cardiac output would be one that is accurate, precise, operator independent, fast responding, non-invasive, continuous, easy to use, inexpensive, and safe. The present paper proposes a new technique for measurement of stroke volume and cardiac output by combining standard cuff-based brachial artery blood pressure data, noninvasive measurements of aortic pulse wave velocity [7], and ultrasonic measurements of aortic luminal cross sectional area [8]. The theoretical feasibility of this alternative technique is explored in three stages. The first stage is an analytical description of the underlying theory of the proposed method, including consideration of tapering of the aortic diameter, and axial gradients in local aortic compliance. The second stage is a test of the validity and accuracy of the method using a numerical model of the aorta and systemic circulation to explore effects of potential confounding variables, including simulated atherosclerosis, nonlinear compliance, and fast heart rates. The third stage is a discussion of the precision of the method based on the propagation of errors from the multiple separate measurements required in the calculation of stroke volume and cardiac output.

### Theory

#### Overview

A well known clinical rule of thumb is that stroke volume is related directly to arterial pulse pressure (that is, systolic minus diastolic pressure) in a given patient, when aortic compliance is constant. The compliance of an elastic tube is the ratio of volume change ΔV to pressure change ΔP when an incremental volume ΔV is introduced. In symbols C = ΔV/ΔP. The dynamic compliance is the instantaneous slope of the volume versus pressure curve. For an aorta of given compliance the stroke volume, SV, is related to pulse pressure PP directly: SV ~ C⋅PP [9–12] if runoff of blood from the aorta is taken into account. Because the compliance is strongly dependent on the diameter of the vessel, the aorta itself and its largest branches are responsible for most arterial compliance seen by blood ejected from the left ventricle. Classically [11, 13] in typical human patients the stroke volume in ml is about 1.5 times pulse pressure in mmHg. That is, in a normal adult human the effective aortic compliance is about 1.5 ml/mmHg. However, in patients with significant abnormalities in arterial compliance, such as atherosclerosis and hypertension, the ratio may be substantially different [10]. The present paper shows theoretically that (1) aortic compliance can be determined from aortic pulse wave velocity and aortic volume, both of which can be measured noninvasively and (2) stroke volume can be determined from systolic and diastolic blood pressure and compliance, even in the presence of pulse reflections and continuous runoff of blood from the aorta, also using completely noninvasive measurements.

#### Estimation of aortic wall compliance

**Figure 1:**
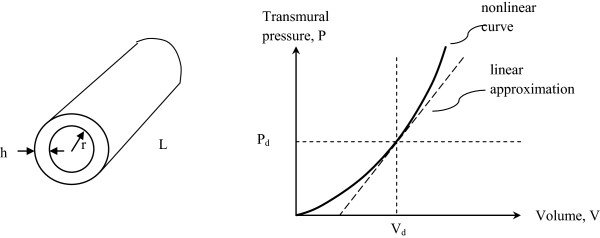
**Ideal elastic tube of length, L, wall thickness, h, and inner radius, r, and its characteristic volume versus pressure function.** The function depends on the elastic properties of the wall material and the difference in pressure across the wall. V_d_ and P_d_ represent reference level, diastolic volume and pressure.

Figure 1 shows an idealized elastic tube of circular cross section and its pressure-volume curve. The compliance of the tube for small incremental additions of volume is

1

where E is the incremental elastic modulus of the vessel wall, h is the wall thickness, and r is the vessel radius [14]. Pulse wave velocity, v, for small volume pulses along an elastic tube containing fluid of mass density, ρ, is governed by the Moens–Korteweg equation,
2a

Solving (2a) for E, we have
2b

and substituting into Equation (1)
3a

in units of , where V is the instantaneous volume of fluid in the tube.

In clinical units of ml/mmHg
3b

Equations (3) state that compliance is simply related to the volume of the vessel, the blood density, and the square of the pulse wave velocity. This relationship is identical to the Bramwell-Hill equation [15], first published in 1922, given the definition of compliance as ΔV/ΔP. Importantly in the present context, aortic volume can be estimated noninvasively using external anatomic landmarks to establish the effective length of the aorta and using ultrasound to measure the mid-level cross sectional area of the aortic lumen [8, 16]. Hence, in principle, aortic compliance can be estimated non-invasively.

#### From compliance to stroke volume

Using the relationship, C = ΔV/ΔP, for the whole aorta, the volume change can be determined from the compliance and the pressure change, under steady state conditions in which the pressure and volume changes are allowed to stabilize. If this state of affairs were strictly true for the aorta, then given a reasonable estimate of compliance, we would have SV ≈ C⋅ΔP, where ΔP is pulse pressure. However, in a real aorta the situation is more complicated for three reasons. First, there is continual runoff of blood from the aorta during and following ejection of blood from the left ventricle, so that the aorta is a leaky compliance. Second, a finite time is required for the pulse wave to travel along the aorta from aortic valve to iliac arteries, hence the pressure may not be the same in all parts of the aorta at the same time, especially during the early part of the pulse wave. Third, there are damped reflections of the pulse wave, so that pressure waveforms in proximal and distal aortic sites differ. Nonetheless, techniques for a more refined estimate of compliance-based stroke volume can be derived as follows.

#### The small animal method

One more exact formulation of the idea that SV ≈ C⋅ΔP takes into account the leakage or runoff of blood from the aorta. In smaller animals by the time just after completion of left ventricular ejection the aortic pulse wave has traveled a distance that is equal to or greater than the length of the aorta. At this time, denoted t_e_, the aortic compliance can be estimated as
4

where SV is stroke volume, CO is cardiac output, t_e_ is the ejection time of the left ventricle, and PP is pulse pressure, which is the pressure step-up in the aorta created by ejection. The term CO t_e_ is a reasonable estimate of the total amount of blood flowing out of the aorta into the peripheral circulation during the time that ejection is occurring. Since cardiac output equals stroke volume multiplied by heart rate, HR, and 1/HR = T, the cardiac cycle length, we can write
5

and solve expression (5) for stroke volume, given estimates of total aortic compliance, pulse pressure, ejection time, and cycle time.

**Figure 2:**
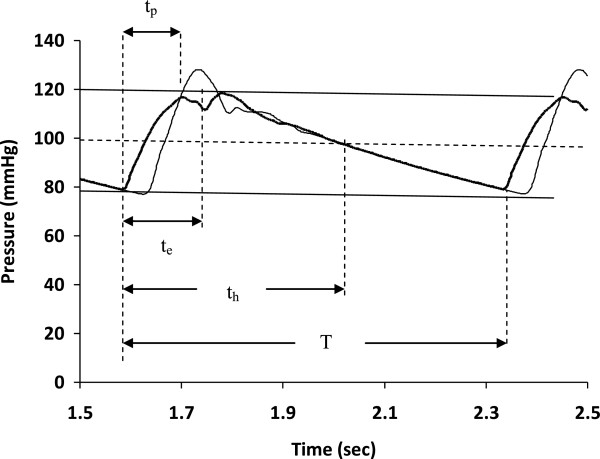
**Sketch of pulse waves in proximal and distal aorta with pulse wave delay and resonance effects.** Four characteristic times are defined for the first pulse wave to the left. The time from onset to peak is defined as t_p_. The ejection time is defined as t_e_. The half time is defined as t_h_. The cycle length or period of the cardiac cycle is defined as T.

There is a problem, however. In larger animals, such as humans, the pulse travel time from the aortic valve to the iliac arteries may be longer than the ejection time under some circumstances, and there is not equilibration of pressures throughout the whole aorta. This effect is especially true in more compliant aortas, in which the pulse wave velocity is slower. In taller adult humans with compliant aortas there may be a substantial delay between the arrival of the pulse in the aortic root and the arrival of the pulse in the abdominal aorta, as shown in Figure 2. As a result the whole aorta does not experience uniform pressure (quasi-steady-state conditions) at the end of left ventricular ejection. Under these conditions the effective aortic compliance that receives the stroke volume is less than the total aortic compliance by a varying amount, and the small animal method will not work. In addition, as shown in Figure 2, there are damped reflections that also complicate pulse contours in the first third of the pulse waveform, so that pressures throughout the whole length of the aorta are not the same.

#### The half time method

To ensure more steady state conditions one can consider the half time, t_h_, defined as the time after the onset of ejection when blood pressure falls to a point halfway between systolic and diastolic pressures. This time is substantially longer than the pulse propagation delay in humans. Further, by this time the oscillations in pulse pressure have been damped out to a large extent, and pressures at all points along the length of the aorta are similar. At time t_h_ the total aortic compliance then comes into play. The halftime can be determined from the pulse contour, as indicated in Figure 2, which can be recorded noninvasively with external pulse pickups [17–19], including those used to measure pulse wave velocity.

At sample time, t_h_, a larger fraction of the stroke volume has already drained from the aorta into the peripheral circulation than was previously estimated at the end of ejection, t_e_. However, as before, a good initial estimate of the leakage rate is the cardiac output, which provides a slight underestimate of the true leakage rate since average aortic pressure is higher during the first part of the aortic pulse contour. Hence, at time point, t_h_, the total aortic compliance can be written as
6

where SV is stroke volume, CO is cardiac output, t_h_ is the half time defined in Figure 2, PP is pulse pressure, and λ is a dimensionless correction factor slightly greater than one. The factor, λ, is the ratio of average blood flow rate exiting the aorta (in mL/min) during time interval 0 to t_h_ from the beginning of the pulse, compared to cardiac output. The term λ⋅CO · t_h_ represents the blood flowing out of the aorta into the peripheral circulation during the time interval t_h_. Since cardiac output equals stroke volume divided by the cardiac cycle length, we can write
7

The correction factor, λ, can be approximated from normal pressure waveforms as about 1.1, or more generally, as shown in Appendix 1, as
8

where , with CVP denoting central venous pressure.

The CVP can be either assumed to be negligible, or if elevated, estimated noninvasively from physical examination of the jugular veins.

Using this approach, one can compute SV and CO from (7) and (8) if total aortic compliance is known.
9

and
10

Equations (9) and (10) represent the “halftime method” for obtaining blood flow from pulse pressure and aortic compliance.

#### Axial tapering and compliance gradients

An obvious limitation of the forgoing analysis is the assumption of uniform diameter and wall thickness for the entire aorta that is implied in Equation (1). Anatomically, it is well known that the aorta tapers slightly in both its internal and external diameters from the level of the aortic root to the aortic bifurcation. Also wall thickness is greater in the thoracic than in the abdominal portions of the aorta.

To assess the impact of such tapering in an analytical way, consider an elastic tube with a constant wall thickness-to-radius ratio, h/r, but with radius, r, varying as a function of length. A constant wall thickness to radius ratio of about 0.12 approximates anatomic reality and makes the mathematical treatment more straightforward. In this case pulse wave velocity  becomes independent of the axial position along the aorta, as long as h/r and the composition of the aortic wall remain the same. Hence Young’s modulus (or the average value of Young’s modulus along the aorta) remains , as before. In this new scenario, however, let the radius, r, of the aorta vary as a function of position, x, from its axial midpoint. For a differential segment of length, dx, along the aorta the compliance using Equation (3) is . To describe anatomically realistic tapering of the aorta let r vary as a linear function of x, such that r(x) = r_0_ – ϵx, where r_0_ is the midpoint of the aorta, with x extending from –L/2 to L/2. Then,
11

Integrating over length, L, we have
12a12b12c

Note that if the total taper, ϵL, is less than r_0_, then for this model, the compliance of the tapered aorta is only slightly greater than that of an un-tapered aorta having the middle value of radius. From the data of Voges et al. [20] we can estimate that ϵL/r_0_ is approximately 6 mm/8 mm, giving about a 4% difference in total compliance with tapering vs. a model of uniform thickness. This is an acceptable error for clinical purposes. If desired, the tapering correction term in parentheses could be taken as a constant for humans, approximately equal to 1.04. Accurate determination of the mid-level aortic cross sectional area, , however, remains important. Also, the issue of nonlinear compliance must be addressed, as shown subsequently.

### Noninvasive data acquisition

#### Pulse wave velocity

At least three different commercial systems are available for measuring aortic pulse wave velocity from external sensors placed over the carotid and femoral arteries [7, 21]. These devices detect the carotid and femoral pulses using external pulse pickups [18, 19] and employ sophisticated algorithms such as cross correlation [22, 23] to determine the time delay between pulses. The time delay is divided into an estimate of effective aortic length to obtain aortic pulse wave velocity. Commercial systems give pulse wave velocities between about 8 and 10 m/sec for typical adult patients having some degree of atherosclerosis [7].

#### Aortic volume

Conventional ultrasound sector scanning can be used to determine aortic cross sectional area in the high abdominal or lower thoracic aorta near its midpoint [8]. One can use external landmarks to estimate the effective length of the aorta, for example as the distance from supra-sternal notch to either anterior-superior iliac spine. For use in Equation (3) the effective length, L, is defined as the length of an un-branched tube having the same total compliance as the natural aorta and its largest (brachiocephalic, carotid, and iliac) branches. The effective length, L, is slightly longer than the anatomic distance from the aortic valve in the chest to the aortic bifurcation in the mid abdomen, including the curvature of the aortic arch. Blood density is essentially constant at 1.03 g/ml. Combining these values in Equation (3) gives a noninvasive estimate of total aortic compliance.

#### Pressures and time intervals

Systolic and diastolic aortic pressures can be determined in the usual way using an external arm cuff or an indwelling arterial catheter. Characteristic time, t_h_, and cycle length, T, can be determined from a noninvasive pulse pickup that records the pulse contour.

In this way cardiac output and stroke volume can be estimated quantitatively from a suite of noninvasive measurements, based on the underlying physics of the aorta. These measurements can be combined to provide estimates of stroke volume by the halftime method. A simple spreadsheet on a laptop computer or application on a smart phone can be programmed to do the calculations automatically, given the input data.

### Testing the proposed methods in a computer model of the circulation

#### Test system

The accuracy of the proposed method can be tested and refined using a computational model of the human systemic circulation. In such a model one can compute pulse wave velocity from proximal and distal pulse waveforms and utilize instantaneous aortic cross sectional area to estimate aortic compliance. In turn, one can compute pulse contour parameters and stroke volume using the halftime method, comparing the results vs. “actual” values in the model. This test system can mimic the details of an idealized aorta in a way that makes it straightforward to investigate possible confounding effects such as, varying stiffness, nonlinear compliance, varying myocardial contractility, and varying heart rate, including both normal and shock-like states.

**Figure 3:**
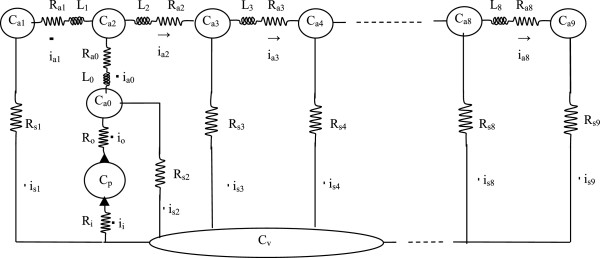
**Mock circulatory system for the numerical model.** Compliances are denoted C. Inertances are denoted L. Resistances are denoted R. Flows are denoted i. Solid triangles indicate one-way heart valves. To simulate left ventricular contraction an external, squeezing pressure, P_ext_(t), having a half sinusoidal waveform is applied to the pump compliance, C_p_, namely P_ext_(t) = P_max_ ⋅ max(0, sin(ωt)), where P_max_ is the peak applied pressure. To create a range of stroke volumes in the model P_max_ is varied from 25 to 175 mmHg. Full details of model construction and operation are provided in Appendix 2.

A simple, 12-compartment numerical model of the aorta and systemic circulation is shown in Figure 3. R_i_ and R_o_ represent the resistances associated with the input and output valves of the left ventricular pump, labeled C_p_. Compliant compartments C_a0_ through C_a9_ represent segments of the aorta and its largest branches. Segment C_a0_ represents the aortic root, and segment C_a2_ represents the aortic arch. Segment C_a1_ represents the brachiocephalic arteries, and segment C_a9_ represents the two iliac arteries. The remaining compliant segments represent the descending thoracic aorta and abdominal aorta.

Small resistances R_a0_, R_a1_, R_a2_, through R_a9_ connect the 10 discrete aortic segments. Resistances R_a1_ and R_a9_ are larger than the other in-line aortic resistance values and represent the damping effects of the more narrow brachiocephalic and iliac arteries and their branches. The in-line aortic resistances were adjusted to give a physically realistic degree of damping to the aortic pulse waveform. The parallel resistances R_s1_ through R_s9_ represent systemic vascular resistance. The values of R_s1_, R_s2_, etc. are over 1000 times larger than the in-line aortic resistances. Included also are inertances, L, representing the inertia of each segment of the aortic blood column. These are computed as the product of fluid density and segment length, divided by segment cross sectional area [24]. The inertances of the venae cavae are assumed to be zero. Their effects are much smaller than those of aortic inertances owing to the much slower blood velocity in the great veins as well as the larger cross sectional areas of the blood columns in the great veins and right atrium. Vascular compliances are denoted by C. The aortic compliances C_a0_ through C_a9_ may take on a range of values from normal to stiff, representing severe atherosclerosis. Numerical values for these and other model parameters are derived in detail in Appendix 2, and are based upon textbook physiology and anatomy. Standard values for the normal circulation are shown in Table 1.

**Table 1:** **Model parameters for a normal adult human**

Parameter	Value	Units	Description
***Resistances***			
Rs1 -- Rs9	10.3	mmHg/(ml/sec)	Local systemic resistance
Ri	0.01	mmHg/(ml/sec)	Pump input resistance
Ro	0.01	mmHg/(ml/sec)	Pump output resistance
Ra0, Ra2 -- Ra8	0.005	mmHg/(ml/sec)	Aortic segment resistance
Ra1, Ra9	0.1	mmHg/(ml/sec)	Carotid and femoral resistance
***Compliances***			
Ca0 -- Ca9	0.13	ml/mmHg	Compliance of an aortic segment
Cv	10	ml/mmHg	Compliance of lumped venous reservoir
Cp	12	ml/mmHg	Pump compliance in diastole
***Dimensions***			
SegLength	5	cm	Aortic segment length
SegRadius	1.5	cm	Initial aortic segment inner radius
VenousVol	2000	ml	Initial volume of lumped veins
PumpVol	200	ml	Initial pump volume
***Knobs***			
frequency	1.3333	Hz	Cardiac frequency (heart rate)
Pmax	120	mmHg	Maximal external pump pressure
dt	0.00001	sec	Time step for numerical integration
startprint	1.5	sec	Time at which output data start being plotted
stopprint	3	sec	Time at which output data stop being plotted
skipnumber	99		Number of calculated points skipped between plotted points
Pinit	10	mmHg	Initial equilibrium pressure of arrested circulation

#### Nonlinear compliance

One obvious difference between the simple test system in Figure 3 and a living subject is that over a wide range of possible physiologic pressures in a given individual dynamic arterial compliance is not constant [14, 25]. Instead the compliance is “nonlinear” because the volume vs. pressure function for arteries is curved over the complete pathophysiologic range. Its slope, the dynamic compliance, varies as a function of pressure. However, a simple modification of the model in Figure 3 can be introduced to mimic the nonlinear characteristics of biological tissues.

#### Mathematical model

The classical nonlinear mechanical properties of tissues such as artery walls have been well characterized by Y. C. Fung [26]. For a tube with walls composed of a classical Fungian biomaterial, the pressure domain analog to the stress–strain relationship would be
13

The reference pressure P_ref_ refers to the pressure at which a fixed experimental value for dynamic compliance is determined, as will be seen. V_0_ represents the zero pressure volume. The use of volume rather than circumferential wall stretch in the exponent is simply accounted for by adjustment of the constant, b. To obtain an expression for nonlinear dynamic compliance, we can recast Equation (13) as,
14

The dynamic compliance is therefore
15

When P = P_ref_ we have , which is the dynamic compliance at the reference pressure, for example, diastolic arterial pressure in the context of the present problem. In turn,
16

as a function of pressure only. Here the reference pressure P_ref_ is analogous to the reference tension T* in Fung’s original model. One can take P_ref_ as the reference pressure at which the aortic compliance is normally determined, such as normal diastolic arterial pressure (80 mmHg) and convert linear compliances, C_ref_, into nonlinear ones, using Equation (16). In this way the numerical model of the mock circulation is easily modified for nonlinear dynamic compliance of the aorta by using expression (16) in place of C_ref_. As instantaneous distending pressure, P, becomes greater than P_ref_, the dynamic compliance becomes less than the nominal value, C_ref_. As instantaneous distending pressure, P, becomes less then P_ref_, the dynamic compliance becomes greater than the nominal value, C_ref_, mimicking the changes in slope of the nonlinear pressure-volume curve in Figure 1.

#### Numerical methods

To make the model circulation go, a positive external, squeezing pressure having a half sinusoidal waveform is applied to the pump compartment. Instantaneous blood flows, volume changes, and pressure changes in all compartments of the model are computed during each discrete time step Δt = 0.00001 sec. These changes are numerically integrated, using the simple Euler method, to give time domain records of instantaneous volumes and pressures in all model compartments, as explained in detail in Appendix 2. The simulations begin with a cold start at the initial vascular volumes and a “mean circulatory pressure” of the arrested circulation of 10 mmHg in all compartments. The left ventricular pressure function is turned on, and at successive time steps, Δt, the following variables are computed to create a marching solution for flows, volumes, and pressures:

vascular flows

volume increments

pressure increments

updated volumes

updated pressures

saved current flows (to compute derivatives)

integral of instantaneous left ventricular outflow (to compute true stroke volume).

This process is iterated over 300,000 time steps to track evolution of the system over a 3 sec test period. The model was solved using Microsoft Visual Basic Macros within a Microsoft Excel spreadsheet, running on a laptop computer, to perform numerical integration. Data are saved for plotting after each 100 time steps (1 kHz sample rate). Steady state pressures are reached after the second heartbeat in this simple system. In addition to pressure and volume waveforms, the aortic radii at each segmental level are computed from the square root of volume/(π⋅length) of each segment as a surrogate for aortic dimensions visualized by ultrasound. Stroke volume during the interval of data recording, corresponding to a whole number, n, of heartbeats, is computed as
17

#### Extraction of parameters from simulated pulse contours

To determine pulse wave velocity for the model circulation in Figure 3 the time interval between upstrokes of the aortic pulse waveforms in segments 2 and 8 was measured directly and divided into the center-to-center distance between the segments. The time difference was measured at a pressure corresponding to diastolic pressure plus 2 percent of the pulse pressure. These points marked the very beginning of the pulse upstroke and corresponded to aortic cross sections at the diastolic level. They are relatively insensitive to reflected wave augmentation in the arterial pressure waveform.

The representative cross sectional area of the aorta, as would be determined by ultrasound, was determined from the average of the diastolic radii of the mid aortic segments (which were quite similar). Blood density was taken as 1.03 g/cm^3^. Total aortic compliance was calculated using Equation (3b), with aortic volume computed as the product of mid-level cross section and the total length of the ten arterial segments. (That is, for the purposes of this simulation the effective length of the aorta was taken as the actual length.) The time interval t_h_, representing the half time was computed from the contour of the function of aortic radius vs. time, working backwards from a time point just prior to the next diastolic point to avoid possible large oscillations after the peak of the pulse. Then stroke volume was computed using the half time formula of Equation (9). Integrated flow across the aortic valve (Equation (17)) was used as the reference, or actual stroke volume.

## Results

### Estimation of aortic compliance

**Figure 4:**
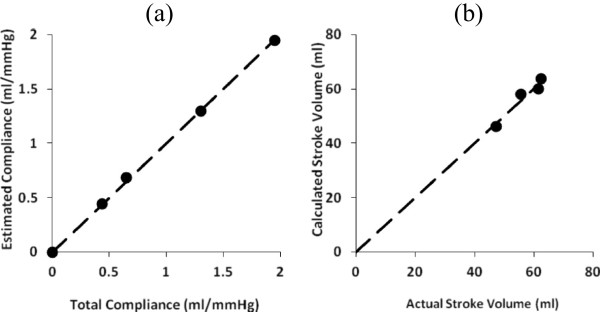
**Estimated total aortic compliance (a) and stroke volume (b) in model circulatory systems with very stiff, stiff, normal, and compliant aortas, plotted as functions of the actual values.** Dashed lines at 45 degrees are lines of identity. Points progressively farther from the origin indicate increasing aortic compliance. The third farthest point from the origin indicates normal aortic compliance. Higher aortic compliance leads to somewhat larger stroke volumes, as expected.

In Figure 4(a) the estimated compliance values based on pulse wave velocity are plotted as a function of actual model compliance over a range of 1/3 to 1.5 times normal for a typical adult human. (Here 1/3 normal compliance corresponds to severe atherosclerosis, ½ normal compliance corresponds to moderate atherosclerosis, etc.) In this first test the model compliance was constant as a function of time and pressure, that is, the volume increased linearly with pressure (a linear compliance model). In this case the true value of model compliance served as a stable reference. The aortic model had constant diameter and material properties along its length. In Figure 4(a) the solid data points represent non-invasively estimated compliance, computed using Equation (3b). The dashed line represents the actual total arterial compliance of the model.

Pulse wave velocities calculated from pulse transit times for 1/3, ½, 1, and 3/2 normal aortic compliance were 1034, 857, 667, and 577 cm/sec respectively. Commercial systems give pulse wave velocities between about 8 and 10 m/sec for typical adult patients [7], who would be expected to have atherosclerosis and relatively stiff, less compliant, aortas. These are quite similar to those in the mock circulation for 1/3 and ½ normal aortic compliance.

Figure (4b) shows the ratio of stroke volume computed from pulse wave velocity and aortic cross section compared to actual stroke volume in the model over a range of aortic compliances from 1/3^rd^ to 1.5 times normal. The halftime method of Equation (9) was used to estimate stroke volume. The agreement for the simple linear compliance model is satisfactory.

### Dealing with nonlinear compliance

**Figure 5:**
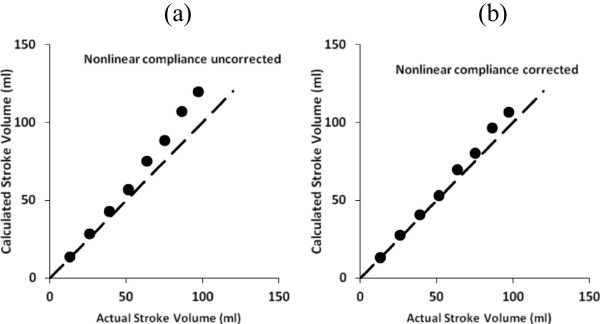
**Estimated stroke volumes in model circulatory systems with nonlinear compliance, plotted as functions of the actual values used as model inputs over a range of peak left ventricular pressure values and consequent aortic pressures. (a)** uncorrected data computed assuming constant aortic compliance **(b)** corrected data for nonlinear compliance in a classical Fungian biomaterial. Heart rate was 80/min. Dashed lines at 45 degrees are lines of identity.

Figure 5(a) shows results from a modified aortic model of constant diameter and nonlinear dynamic compliance, computed at each time step of the numerical integration using Equation (16). As before, non-invasively estimated compliance was computed using equation (3b) from pulse wave velocity and model aortic volume without any correction for nonlinearity. Stroke volume was computed using Equation (9) over a range of peak left ventricular pressures from 25 to 175 mmHg, which produced actual stroke volumes ranging from roughly one quarter to twice normal. Figure 5(a) shows the estimated stroke volume computed from pulse wave velocity and aortic cross section compared to actual stroke volume in a typical model with normal aortic compliance. The estimated values slightly overestimate true stroke volume, especially at higher pulse pressures. Here the pulse wave velocity method is giving diastolic compliance, but the average compliance of the aorta over the time that blood is draining into the periphery is less, because of the nonlinear effect. In turn, the observed pulse pressure is greater than expected for the diastolic compliance level. Stroke volume estimates based on the product of observed pulse pressure and observed diastolic compliance are greater with nonlinear compliance, especially at higher pulse pressures.

The overestimate can be corrected, in large part, as shown in Figure 5(b), using the following nonlinear compliance correction for pressures in units of mmHg,

18a

which is derived in detail in Appendix 3. In Equation (18a) SV’ indicates the computed stroke volume estimate in the presence of nonlinear compliance characteristics typical of a classical Fungian biomaterial. SV indicates the stroke volume estimate of Equation (9) that is computed assuming constant compliance, PP is the pulse pressure, and DBP is the diastolic blood pressure, both in units of mmHg. The function ln(x) is the natural logarithm of x. In the limiting case for small values of pulse pressure, PP, one can use the series approximation for ϵ < < 1
18b

This form of expression (18a) shows that for small pulse pressures the correction factor approaches 1.00. For increasingly larger pulse pressures the correction factor becomes progressively less than 1.00.

When the correction ratio (18a) is applied to the data in Figure 5(a) the results in Figure 5(b) are obtained. The systematic overestimation of stroke volume at larger pulse pressure is largely eliminated. The correlation coefficient (r^2^) for estimated vs. actual stroke volume in Figure 5(b) is 0.998.

### Varying heart rate

**Figure 6:**
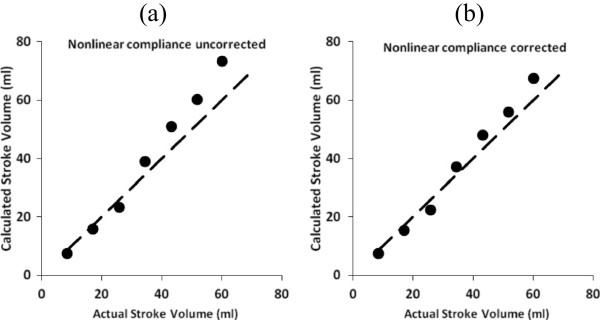
**Estimated stroke volumes in model circulatory systems with nonlinear compliance, plotted as functions of the actual values used as model inputs over a range of peak left ventricular pressure values and consequent aortic pressures. (a)** uncorrected data computed assuming constant aortic compliance **(b)** corrected data for nonlinear compliance in a classical Fungian biomaterial. Heart rate was 120/min. Dashed lines at 45 degrees are lines of identity.

Figure 6 shows noninvasively estimated stroke volume vs. actual stroke volume for a fast heart rate (120/min) as might occur in patients with heart failure or shock-like states. The correlation coefficient (r^2^) for estimated vs. actual stroke volume after nonlinear compliance correction was 0.983.

## Discussion

A global emphasis on cost effectiveness in medicine has made it timely to revisit the issue of noninvasive measurement of cardiac output and stroke volume, which remains an open problem in biomedical engineering. Classical standard methods, inducing the Fick and indicator dilution techniques, require centrally placed right heart catheters to sample mixed venous blood or to gain access to the combined circulatory flow in the pulmonary artery. Pulse contour methods frequently require at least one invasively obtained calibration value, which should be repeated if mean aortic pressure changes, owing to the nonlinear compliance of the aorta. Bio-impedance methods [6, 27] are fully noninvasive, but somewhat difficult to calibrate, although novel front-to-back electrode placements can help to give a fully noninvasive estimate of cardiac ejection fraction, if not absolute stroke volume [28]. The present paper proposes a new strategy, based on an old equation [15] that allows for fully noninvasive estimates of the absolute value of stroke volume, and in turn, cardiac output. The results show that it is at least theoretically possible to obtain accurate estimates of stroke volume in human adults over a wide range of aortic stiffnesses, heart rates, and ejection fractions. Moreover, the calculations can account gracefully for nonlinear aortic compliance. Unlike other non-invasive techniques for estimating cardiac output, which have been well reviewed [6, 29], the present method does not involve proprietary algorithms. The validity and assumptions of the approach are fully open to evaluation. Modifications and refinements can be made easily in response to future laboratory and clinical experience. Of course, pulse wave velocity based estimates of stroke volume would be precluded in patients with severe cardiac arrhythmias and in patients with intra-aortic balloon pumps. In such patients a pulmonary artery catheter may be justified.

### Propagation of errors from multiple measurements

A potential weakness of the proposed technique is the precision with which pulse transit time and pulse wave velocity can be measured, since the computed compliance depends on the square of the pulse wave velocity. There is also potential error in the cross sectional area of the aorta near its midpoint, as measured by ultrasound, as well as in externally measured systolic and diastolic blood pressures and in the cardiac cycle length and halftime values.

To analyze error compounding in the proposed technique one can estimate the statistical propagation of errors in the formula for cardiac output
19

The principal uncertainties are in the aortic cross section, A_mid_, the effective length estimate, L, the pulse pressure, PP, the pulse wave velocity, v, and the lumped timing factor (T ‒ λt_h_). One can explore random variation in calculated cardiac output as a function of the random variation in these five quantities, especially the squared pulse wave velocity term. This analysis is analogous to the following statistical problem. Consider uncorrelated random variables X, Y, Z, U, V, and S such that
20

Let  denote the mean of random variable, X, σ(X) denote the standard deviation of X, and σ^2^(X) denote the variance of X, and similarly for the other random variables. Taking natural logarithms,
21

For uncorrelated variables that are added or subtracted the variances add, such that
22

We can use the relationship for the variance of the natural logarithm of a random variable (Appendix 4)
23

A good index of variability in a positive random variable is the coefficient of variation, defined as the standard deviation divided by the mean. For the composite random variable, S,
24

This result is similar to the general rule for the propagation of errors in chemical measurements [30]. For example if X, Y, Z, U, and V each have a coefficient of variation, ϵ, then
25

And if ϵ = 0.05, then . If there are 5% errors each in the aortic cross section, pulse pressure, aortic length estimate, lumped timing factor, and pulse wave velocity, then the expected variation in computed cardiac output would be 14 percent. The 95% confidence limits, roughly two standard deviations, would be 28 percent of the mean value. This estimate is within the clinically acceptable limit of 30 percent variation, proposed by Critchley and Chritchley [31].

The potential lack of precision in noninvasively estimated cardiac output must be compared with the lack of precision in established “gold standard” techniques, which is also non-trivial [31]. The Fick method, for example is an aggregate of independent measurements of oxygen uptake and arteriovenous oxygen difference. The indicator dilution method requires independent measurements of the amount of dye injected and the area under the dilution curve compared to baseline. The estimate using Equation (25) is similar to that of other alternative methods of measuring cardiac output as reviewed by Geerts [6], in which coefficients of variation ranged from 5 to 15 percent. Hence, then proposed noninvasive approach would appear to have potential precision similar to that of existing methods with fewer drawbacks and complications.

More precise measurements of the component variables, would lead to correspondingly better estimates of stroke volume. An important opportunity for increasing precision is the ability to sample and average data for 10 to perhaps 100 heartbeats using an automated data collection system. Random variations can be reduced substantially by such averaging. It remains as a challenge to experimentalists to create practical systems with sufficient precision for practical, clinical implementation.

In discussing the overall validity of the proposed method, however, it is important to distinguish between errors in accuracy introduced by constant biases and true random biological variation. Constant systematic biases may be introduced into the calculations, but are less troublesome than unpredictable random variations. For example, in determining the effective aortic length, L, based on superficial landmarks, it may well be that the actual aortic length is, on average, say, 10 percent more than the landmark based measurement, with individual patients varying somewhat about this value. In determining the precision of the method the constant average bias is not important. Clinicians can easily adapt to a constant 10 percent overestimate of stroke volume. Changes over time in the management of an individual patient remain easy to recognize and interpret. Further, with experience such constant biases can be reduced to acceptable levels.

There are alternative solutions to the problem at hand. The lack of evidence for the cost effectiveness of invasive hemodynamic monitoring using a pulmonary artery catheter [32] has prompted commercialization of systems to track changes in stroke volume by pulse contour analysis. These systems have been well reviewed [6, 13, 29, 33]. The PiCCO System (Pulsion Medical Systems, Munich, Germany) derives stroke volume estimates from the diastolic to systolic integral of the pulse contour, which is calibrated by invasive thermal dilution measurements, using a dedicated catheter typically placed in the femoral artery. The calibration must be repeated every time there is a significant hemodynamic change. The LiDCO plus system (LiDCO, Cambridge, UK) uses a proprietary pulse pressure algorithm (PulseCOTM) based on the change in power in the arterial tree and repeated lithium dilution for calibration. These systems allow continuous monitoring of changes in hemodynamics but still require placement of invasive catheters for calibration. The Flotrac system (Edwards Lifescience, Irvine, CA, USA) incorporates a specific transducer that may allow calibration using a non-central arterial line for accurate waveform acquisition with calibration based on nomograms using the age, weight, and sex of the patient to estimate compliance. The Flowtrac algorithm uses a multivariate scaling parameter reflecting the effects of vascular tone on pulse pressure that is computed from a polynomial equation. None of these systems is truly noninvasive.

A completely noninvasive approach has been described classically by Huntsman and coworkers [16], who used ultrasound to measure the volume of blood moving through the ascending aorta during systole. The blood velocity was calculated from Doppler measurements and the cross sectional area was measured in A mode or M mode. Stroke volume was calculated as V x ET x CSA, where V = the spatial average blood velocity in the aorta during systole, ET = ejection time and CSA = the cross-sectional area of the lumen. Of course the roughly 7 percent of stroke volume flowing into the coronary arteries would be missed by this method. The approach appears to be relatively dependent on the skill of the operator, requiring 10–15 minutes for the initial diameter assessment [16] by unhurried examination of the anatomy and repeated diameter determinations. In addition, the first velocity measurement may take as long as 5 minutes. Nonetheless, this classic paper shows the clinical feasibility of fully noninvasive measurements of stroke volume and cardiac output.

## Conclusions

The present analytical and numerical modeling exercise suggests that a completely noninvasive pulse wave velocity based method for measuring stroke volume can in principle be accurate, despite pulse wave reflections within the aorta and nonlinear vascular compliance. That is, it can give the true value in the absence of noise. Follow-on clinical studies are needed to determine if the real-world precision of this approach is sufficient for monitoring the circulatory status of critically ill patients and if the method is practical and feasible in real-world clinical settings.

## Appendix 1: estimation of λ -- flow during the halftime interval vs. cardiac output

**Figure 7:**
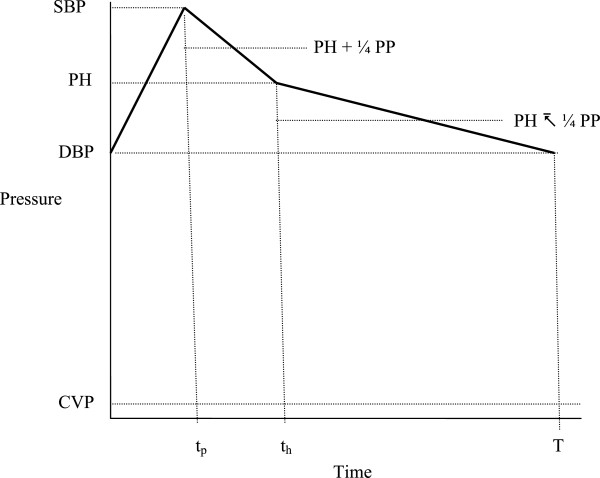
**Piecewise linear approximation to a blood pressure waveform with significant points and levels.**

For mathematical convenience one can model the pulse wave as a piecewise linear function rising from the diastolic point to the systolic point, then falling to the halftime point, and then falling to the next diastolic point, as shown in Figure 7.

### Define

SBP as systolic arterial pressure

DBP as diastolic arterial pressure

PP as pulse pressure (systolic minus diastolic)

PH as halftime pressure (the average of systolic and diastolic pressure)

CVP as central venous pressure

t_p_ as time to peak from the diastolic point

t_h_ as time from diastolic point to halftime point

T the cardiac period or cycle length

PH’ as PH minus CVP

Mean systemic perfusion pressure (mean aortic pressure minus CVP) over the whole cardiac cycle, which is proportional to mean flow or cardiac output, is the area under the pressure pulse curve, divided by its base, or
26

The corresponding mean systemic perfusion pressure during the halftime interval from 0 to t_h_ is
27

Expanding the terms, cancelling, and dividing by PH’ gives,
2829

Equation (28) states that because of the shape of the blood pressure waveform, the mean perfusion pressure during the first part of the pressure pulse is slightly greater than PH’ (the average of systolic and diastolic perfusion pressures). Equation (29) states that because of the shape of the blood pressure waveform, the overall mean perfusion pressure is slightly less than the average of systolic and diastolic perfusion pressures. The desired ratio of perfusion during the halftime interval to cardiac output or average perfusion over cycle length, T, is the ratio of Equations (28) and (29) or
30

for the piecewise linear approximation. For computation with 31

For non-invasive estimates the terms t_p_ and t_h_ can be determined from the time domain output of an external pulse pickup. Systolic and diastolic arterial pressures can be determined noninvasively as well by the usual cuff method and CVP by physical examination of the jugular veins.

## Appendix 2: systemic circulation model

Text Figure 3 illustrates a simple, 12-compartment numerical model of the circulatory system, for which numerical values for inertances, resistances, and compliances are determined as follows.

### Inertances

Inertances [24], L, of each segment of the aorta are computed from blood density, ρ ≈ 1.03 g/ml, segment length, s, in cm, and segment internal cross section, A, in cm^2^ as
32

in units of (dynes/cm^2^)/(ml/sec^2^) or
33

in units of mmHg/(ml/sec^2^), where instantaneous cross sections, A, are computed from segmental volume divided by the fixed segment length.

### Resistances

Systemic vascular resistance is estimated as normal mean arterial pressure divided by cardiac output or 95 mmHg/(5 L/min) = 95 mmHg/(83.3 ml/sec) = 1.14 mmHg/(ml/sec) in total. Each of the 9 parallel systemic vascular resistances in text Figure 3 was 10.3 mmHg/(ml/sec) or 9 times the total resistance.

Segmental resistances to axial blood flow along the aorta, R_a0_ and R_a2_ through R_a8_, are more than three orders of magnitude smaller than corresponding vascular resistances to drainage of blood from the aorta into the lumped venous compartment. These segmental resistances were initially estimated using Poiseuille’s law and then increased empirically to provide a physiologically realistic amount of damping of the arterial pulse waveform in the mock circulation. Final values for R_a0_ and R_a2_ through R_a8_ were 0.005 mmHg/(ml/sec) and remained constant for all simulations. These aortic resistances are equal to each other (no tapering unless otherwise specified). In addition, the end resistances R_1_ and R_9_ are further increased to 0.1 mmHg/(ml/sec) to model the increased resistance of narrowing vessels in the carotid and femoral arterial trees. Similarly, the pump inflow and outflow resistances, R_i_ and R_o_, are taken as small values = 0.01 mmHg/(ml/sec).

### Compliances

Using 280 msec as a normal ejection time [34], normal compliance of the aorta is estimated as 83.3 ml/40 mmHg multiplied by (1 – t_e_/T) (text equation (5)) or 2.08 * (1–0.28/0.75) = 1.3 ml/mmHg. For ten equal aortic segments (no tapering) the compliance of each is 0.13 ml/mmHg normally. Total venous compliance is much larger than aortic compliance and is taken as 10 ml/mmHg. A nominal value for diastolic pump compliance is taken as 60 ml/5 mmHg = 12 ml/mmHg or stroke volume divided by the difference between end-systolic and end-diastolic pressure in the left ventricle.

### Vascular dimensions

For this initial model the length of each aortic segment is taken as 5 cm and the radius of the aorta is taken (uniformly, unless otherwise specified) as 1.5 cm. The initial volume of the venous reservoir is 2000 ml. The initial volume of the heart pump in an arrested circulation is modeled as 200 ml.

### Pump function

To make the model go an external, squeezing pressure, P_ext_(t), is applied to the pump compartment having a half sinusoidal waveform, namely
34

where P_max_ is the peak applied pressure. To create a range of stroke volumes in the model P_max_ was varied from 25 to 175 mmHg.

Pump inflow, i_i_, represents ventricular filling. Pump inflow is given by the expression
35

Pump output flow, i_o_, represents ventricular ejection.
36

Here the max() functions simulate the effects of the inflow and outflow valves, permitting one-way flow only through the pump.

During a discrete time step, Δt, the changes in internal pump volume and internal pump pressure are given by
37

and, using the derivative of external pump pressure in Equation (34),
38

The cosine function in (38) represents the time derivative of P_ext_(t). Instantaneous pump volume and instantaneous pump pressure as functions of time are determined by numerical integration of expression (37) and expression (38).

### Segmental flows

As shown in text Figure 3, the first aortic segment, indexed a0, represents the ascending aorta, Segment a1 represents the brachiocephalic arteries, and segment a2 represents the aortic arch. Thus flow proceeds from the left ventricle into the ascending aorta (a0), then to the aortic arch (a2), and splits between the brachiocephalic vessels (a1) and the descending thoracic aorta (a3), with flow continuing down the aorta to segment a9. At each time step in the simulation the segmental flows are computed using Ohm’s law as
39a39b39c39d

and so on. Systemic flows are given by
40a40b

representing coronary artery blood flow as shown in text Figure 3, and
40c40d

### Compartmental volume and pressure changes

At each time step of the simulation the changes in fluid volume and pressure in each compartment are computed next as
41a41b41c

in keeping with the plumbing and notation of text Figure 3. Continuing, we have
41d41e

and so on, followed by
42

### Flow derivatives

Flow derivatives, used to calculate inertial effects are then computed from the current and saved last instantaneous flow values, divided by Δt, as follows:
43

### Computational formulas for instantaneous aortic flows

Substituting these discretized values (43) into the expressions (39) for flow gives, for example,
44a44b44c45

Expressions of this form (45) are used as computational formulas for instantaneous aortic flows i_a0_, i_a1_, … through i_a8_.

## Appendix 3: nonlinear compliance correction

For an aorta having a wall composed of a classical Fungian biomaterial the volume vs. pressure relationship is given by previously derived text Equation (14), namely
46

where the constant, , as previously explained. Here P_ref_ is a reference pressure, close to normal diastolic pressure, where a standard value of compliance at normal physiologic pressure, P_ref_, would be defined in the equation for the nonlinear compliance model. This standard value of dynamic compliance at the reference pressure from Text Equation (16) is . Hence, the volume versus pressure curve is given by
47

and the difference in volume between two steady state pressures P_1_ and P_2_ is
48

For the halftime method of text Equation (9) we have , which represents the volume of blood added to the aorta during the last left ventricular ejection that still remains within the aorta at halftime, t_h_. Therefore, using the primed variable, SV’, to denote the nonlinear condition, and Equation (48) for V_1_ – V_2_, where V_1_ is the halftime aortic volume and V_2_ is the diastolic aortic volume, we have
49

Using pulse wave velocity to estimate compliance at the prevailing diastolic blood pressure, the estimated compliance for the nonlinear compliance model would be
50

so that 2 C_ref_P_ref_ = C_est_(DBP + P_ref_) and using Equation (49),
51

The halftime method gives an uncorrected value for stroke volume based on text Equation (9), assuming constant compliance C_est_, of
52

Dividing Equations (51) and (52), the ratio of stroke volume with nonlinear aortic compliance to that calculated assuming constant compliance is
53

Equation (53) gives a small correction for the presence of nonlinearity. Taking the reference pressure P_ref_ as 80 mmHg or normal diastolic blood pressure,
54

In the limiting case for very small values of pulse pressure, PP, one can use the series approximation for ϵ < < 1
55

As expected, for extremely small pulses the effect of nonlinearity is negligible. For increasingly larger pulses the correction factor becomes progressively less than unity.

For blood pressure of 120/80 Equation (54) gives 0.942 and Equation (55) gives 0.938. For blood pressure 180/40 Equation (54) gives 0.473 and Equation (55) gives 0.708. Hence, in general, and especially for higher pulse pressures, one must use the exact logarithmic expression of Equation (54).

## Appendix 4: the delta method

The probable error method or delta method [30, 35] may be used to approximate variances of functions of random variables. If X is a random variable with mean μ and variance σ^2^, the variance σ^2^(f(X)) ≈ σ^2^(X) (f′(μ))^2^ where f′(X) is the first derivative of function f(X) with respect to X. To appreciate the approximation one can visualize the function f(X) as a graph with a tangent of slope f′(μ) at point (μ, f(μ)). By deduction from such a graph, it follows that the standard deviation of f(X) is approximately f′(μ) times the standard deviation of X, as long as f′(X) does not change greatly over the range of X. For the case of f(X) = ln(X), the delta method gives .
